# Melatonin mitigates dichlorvos-induced derangements in the testes and epididymis of male Wistar rats

**DOI:** 10.1590/1984-3143-AR2024-0098

**Published:** 2026-02-09

**Authors:** Olumide Samuel Ajani, Ekundayo Stephen Samuel

**Affiliations:** 1 Department of Theriogenology, Faculty of Veterinary Medicine, University of Ibadan, Ibadan, Oyo State, Nigeria; 2 Department of Veterinary Physiology and Biochemistry, Faculty of Veterinary Medicine, University of Ibadan, Ibadan, Oyo State, Nigeria

**Keywords:** dichlorvos, melatonin, testes and epididymis, histology, morphometry

## Abstract

Dichlorvos (DDVP) is a widely used organophosphate insecticide. The reports of its toxicity and induction of oxidative stress have warranted the search for an antidote. Melatonin (MLT), a hormone found naturally in the body is known to mitigate oxidative stress. Therefore, the present study aimed to investigate the protective effects of melatonin on dichlorvos-induced toxicity in the testes and epididymis. Sixty (60) 10 weeks of male Wistar rats (160 ± 10g) randomly grouped into four (n=15, A-D) were used and treated as follows: A (Control): corn oil (1 mL/kg body weight), B: MLT (10 mg/kg), C: DDVP (1.6 mg/kg), and D: DDVP and MLT. Except for the MLT intra-peritoneal treatment, other test samples were administered orally for 45 days. Histomorphometry and histological examinations were conducted on the testes and epididymis 24h, 14 days, and 45 days after treatment. There was a significant decrease in the epithelial length of the epididymis of rats treated with DDVP at 24h and 45 days post-treatment compared to the control. MLT significantly increase the mean epididymal epithelial length at 24h and 45 days post-treatment in the co-treated group. The DDVP-induced significant reduction in the testes’ luminal diameter was reversed by MLT in the co-treated group. MLT, DDVP (45th-day treatment), and DDVP+MLT (14th and 45th-day treatment) increased the epididymal luminal diameters significantly compared to the control. Meanwhile, DDVP altered both the testicular and epithelial architectures and caused atrophy of the seminiferous tubules and spermatogenic arrest with atrophy of epithelial tubules, especially following 14- and 45-day treatment. These lesions were reversed in the co-exposed rats by MLT. In conclusion, melatonin mitigates the derangements induced by dichlorvos in the testes and epididymis.

## Introduction

Organophosphate (OP) pesticides are among the most widely used synthetic chemicals for controlling pests. The main mechanism of organophosphate pesticides is inhibition of acetylcholinesterase (AChE), which hydrolyses acetylcholine (ACh) in cholinergic synapses and at neuromuscular junctions (Verma et al., 2009; Kalender et al., 2012; Tsai and Lein, 2021). The inhibition of AChE by organophosphate pesticides causes the accumulation of acetylcholine at cholinergic synapses, with over-stimulation of muscarinic and nicotinic receptors (Ben Salem et al., 2015). Pesticides damage the lipoidal matrix in cells, generate reactive oxygen species, and promote oxidative stress that interferes with the normal functions of the reproductive system (Joshi et al., 2003; Uzunhisarcikli et al., 2007; Park et al., 2024).

Dichlorvos (2,2-dichloroethenyl dimethyl phosphate, DDVP) is an organophosphate insecticide that has been widely used in the agricultural industry and domestically in the control of flies, mites, caterpillars, and other insects (Binukumar and Gill, 2010; Okoroiwu and Iwara, 2018). Despite these uses, dichlorvos toxicity following its ingestion, inhalation, or absorption has been reported in the skin, lung, liver, intestine, brain, and testes (Mathur et al., 2000; Binukumar et al., 2010; Aditya et al., 2012; Wang et al., 2013; Zhao et al., 2015; Saka et al., 2024; Zhang et al., 2025). Exposure to DDVP causes symptoms such as weakness, headache, salivation, vomiting, diarrhoea, skin irritation, and toxicity in the reproductive system (Okamura et al., 2005; Oral et al., 2006; Ezeji et al., 2015; Saka et al., 2024). However, several reports have suggested that organophosphate pesticides, including DDVP, induce DNA damage and oxidative stress; with the formation of excessive reactive oxygen species (Ben Salem et al., 2015; Prathiksha et al., 2023). Oxidative stress has been implicated in the pathogenesis of several disease conditions following exposure to organophosphate pesticides (Ben Salem et al., 2015; Saka et al., 2024). The body has developed several defence mechanisms against oxidative damage (Jomova et al., 2024).

Melatonin (N-acetyl-5-methoxyindolamine) is a hormone found naturally in the body and a derivative of tryptophan, an essential amino acid for mammals. The report has it that melatonin is uniquely synthesised and secreted by the pineal gland in vertebrates (Reiter, 1991; Andronachi et al., 2025) and functions in defence of environmental stresses via free radical scavenging (Tan et al., 2003; Chitimus et al., 2020). In addition to the role of melatonin on circadian rhythms, sleep induction, regulation of seasonal reproduction and immune enhancement (Reiter, 1991), melatonin acts as an antioxidant via free radical scavenging (Reiter et al., 2016; Galano et al., 2018). Melatonin increases the efficiency of mitochondrial oxidative phosphorylation, reduces electron leakage, and augments the efficiency of other antioxidants (Reiter et al., 2003; Reiter et al., 2016). Melatonin supplementation prevents brain and cardiac injury (Jiki et al., 2018; Hu et al., 2023), shows higher potency than vitamins C and E in protecting tissues from oxidative damage, and does not alter vascular function in healthy normotensive adults (Tan et al., 2003; Ramos-Gonzalez et al., 2023). Despite the various documented uses of melatonin, its potential in mitigating organophosphate toxicity has not been elucidated. Therefore, the present study was designed to investigate the protective effects of melatonin on dichlorvos-induced toxicity in testes and epididymis of male Wistar rats.

## Methods

### Experimental animals

Sixty (60) 10 weeks of male Wistar rats weighing 160 ± 10g were used in this study. The animals were obtained from the Experimental Animal House of the Faculty of Veterinary Medicine, University of Ibadan, Ibadan, Nigeria. The animals were kept in cages (60 × 60 × 50 cm). All animals were kept under controlled conditions of temperature (25 ± 2.0°C), relative humidity (50 ± 15%), and normal photoperiod (12-hour light and 12-hour dark). The animals were fed on a standard rat diet (commercial pellet), and water was provided *ad libitum*. During this research work, the regulations and principles of the care and use of experimental animals were strictly adhered to. The well-being of the animals, such as proper housing, standard feeding, humane handling and disease prevention treatment as per the guidelines as approved (Ref: UI-ACUREC /17/0070) by the Animal Care, Use and Research Ethical Committee, University of Ibadan, Nigeria.

### Chemicals

Dichlorvos (DDVP, 98% purity) and Melatonin (MLT) were purchased from Sigma-Aldrich Co. (St Louis, Missouri, USA). 1.6 mg/kg body weight oral dose DDVP, corresponding to 1/50 LD50 was used (Okoroiwu and Iwara, 2018). All other chemicals used in this study were of the highest available grades.

### Experimental protocol

The sixty (60) adult male rats were randomly assigned into four groups of 15 animals each as follows:

- Group A: (Control)- Rats were orally administered corn oil (1 mL/kg body weight)
- Group B: (MLT-treated rats) received 10 mg/kg body weight melatonin intra-peritoneally
- Group C: (DDVP-treated rats): Dichlorvos was administered orally at 1.6 mg/kg
  • Group D: (DDVP+MLT-treated rats): Rats were orally exposed to DDVP (1.6 mg/kg) and MLT (10 mg/kg) intra-peritoneally.

All treatment**s** lasted 45 days to evaluate the effect of DDVP through a complete spermatogenic cycle, which takes approximately 45 days in rodents (Chitra et al., 2003). Twenty-four (24h) (acute) after the first treatment, the rats were weighed, and 5 rats from each group were euthanised according to Chitra et al. (2003), and subsequently samples were collected at 24h, 14 days and 45 days post-treatment for histological examinations and histomorphometry analysis. The choice of doses/duration of treatment of DDVP and MLT, as well as the selected time points (24 h, 14 days and 45 days) for treatment and sample collection were according to previous reports for both test samples with slight modification (Dirican and Kalender, 2012; Olukole et al., 2019; Olukole et al., 2020).

### Histological examination of the testis and epididymis

Following animal euthanasia, a mid-caudoventral abdominal incision was made with sterilised scissors to separate the testes from the epididymis as described by Oyeyemi and Fayomi (2011). The testis was observed for any evidence of gross lesion and weighed, subsequently. The gonadosomatic index (GSI) was estimated [(testis weight/body weight) × 100]. The excised testicular samples were stored in bouin solution for tissue sectioning. Sections 5-μm thick were stained with haematoxylin and eosin (H&E) after embedding in paraffin and subsequent histological examination using methods described by Chayen et al. (1973). Five histological fields per section of stained testes and epididymis from three different rats randomly chosen per group were examined under the microscope (Olympus Bx63, Olympus Corporation, Tokyo) at ×400 magnification and interpreted by an experienced pathologist. All other approaches for the evaluation of testicular toxicity were performed as described by Creasy (2003).

### Histomorphometry of the testes

Histomorphometry was done using ToupeView 3.7® Software. The following measurements were taken: seminiferous (testes) tubular diameter, epididymal luminal diameter, and epididymal epithelial height, using AmScope® camera fitted to an Olympus® microscope. For each parameter, ten measurements were collected using a calibrated eye-piece micrometre (Graticules Optics Ltd, Tonbridge Kent, UK).

### Statistical analysis

The data generated was analysed using the Test of Homogeneity of variance, multiple comparisons and Analysis of Variance (One-Way ANOVA). SPSS Version 15 for Windows (SPSS Inc, 2006) and Microsoft Excel Professional Plus (Microsoft Corporation, 2010) were used to carry out all procedures. Significance was set at p< 0.05.

## Results

The results of the parameters illustrated in the study are presented for the control, melatonin, dichlorvos, and dichlorvos and melatonin-treated rats, respectively. Tables 1 and 2 show that there were no significant changes (p>0.05) in the gonadosomatic index of the testes and epididymis, respectively of rats across the treatment groups at 24h, 14-, and 45 days post-treatments.

**Table 1:** Gonadosomatic index of the testes in different treatment groups.

PERIOD (Post-Treatment)	GONADOSOMATIC INDEX OF TESTES (%)	CONTROL	MLT	DDVP	DDVP+MLT
24h	Left	1.196±0.2521a	1.444±0.1045^a^	1.212±0.2652^a^	1.280±0.1030^a^
	Right	1.144±0.2677^a^	1.386±0.05857^a^	1.170±0.2102^a^	1.216±0.07570^a^
14 days	Left	0.9120±0.3938^a^	1.268±0.2068^a^	1.160±0.1271^a^	1.118±0.07320^a^
	Right	0.9080±0.3507^a^	1.228±0.1539^a^	1.120±0.09083^a^	1.083±0.09535^a^
45 days	Left	1.094±0.1740^a^	1.130±0.1400^a^	1.116±0.1381^a^	1.132±0.1047^a^
	Right	1.108±0.1112^a^	1.112±0.1372^a^	1.092±0.1417^a^	1.092±0.08526^a^

Values are reported as mean ± SD. ^a^ Means in the same row with different superscript are significant from control (p<0.05).

**Table 2:** Gonadosomatic index of the epididymis of rats in different treatment groups.

GONADOSOMATIC INDEX OF EPIDIDYMIS (%)	CONTROL	MLT	DDVP	DDVP+MLT
24h post-treatment	0.528±0.1718a	0.628±0.07887^a^	0.46±0.1173^a^	0.536±0.04930^a^
14 days post-treatment	0.344±0.1620^a^	0.4625±0.08884^a^	0.438±0.05805^a^	0.415±0.03873^a^
45 days post-treatment	0.486±0.04980^a^	0.458±0.05630^a^	0.442±0.06797^a^	0.448±0.05450^a^

Values are reported as mean ± SD. ^a^ Means in the same row with different superscript are significant (p<0.05).

Similarly, no significant changes (p>0.05) were observed in the teste’s length and diameter of rats in all the treatment groups at 24h, 14-, and 45 days post-treatment (Tables 3 and 4). However, DDVP reduced the left testis diameter significantly after 24h treatment (Table 4).

**Table 3:** Gross testes length of rats in treatment groups.

PERIOD (Post-Treatment)	GROSS TESTES LENGTH (mm)	CONTROL	MLT	DDVP	DDVP+MLT
24h	Left	18.34±1.640a	19.33±2.449^a^	16.56±0.9247^a^	17.65±1.004^a^
	Right	18.72±2.319^a^	19.05±1.595^a^	16.69±1.063^a^	17.13±1.421^a^
14 days	Left	14.77±3.587^a^	17.11±1.521^a^	16.97±1.375^a^	16.81±1.685^a^
	Right	14.44±3.179^a^	17.24±1.120^a^	17.07±0.8872^a^	16.77±1.010^a^
45 days	Left	15.84±0.9964^a^	16.09±0.8803^a^	16.62±0.9837^a^	16.37±0.8928^a^
	Right	16.53±0.7816^a^	15.97±0.8631^a^	16.16±0.7603^a^	16.55±0.8592^a^

Values are reported as mean ± SD. ^a^ Means in the same row with different superscript are significant from control (p<0.05).

**Table 4:** Gross testes diameter of rats in treatment groups

PERIOD (Post-Treatment)	GROSS TESTES DIAMETER (mm)	CONTROL	MLT	DDVP	DDVP+MLT
24h	Left	9.768±1.038a	9.388±0.9516^a^	8.200±0.5881b	8.798±0.5494^a^
	Right	10.01±1.032^a^	9.350±1.437^a^	8.324±0.7245^a^	8.758±0.8044^a^
14 days	Left	7.872±2.038^a^	9.130±0.5547^a^	8.502±0.3320^a^	8.118±0.8473^a^
	Right	8.016±1.825^a^	8.888±0.4492^a^	9.080±0.6680^a^	8.100±0.3896^a^
45 days	Left	8.692±1.058^a^	9.070±0.3201^a^	8.514±0.5139^a^	8.648±0.4855^a^
	Right	8.646±0.9058^a^	8.780±0.2289^a^	8.732±0.5036^a^	8.758±0.2831^a^

Values are reported as mean ± SD. ^ab^ Means in the same row with different superscript are significant from control (p<0.05).

Treatment with DDVP caused a significant decrease (p<0.05) in the testes luminal diameters of rats as compared to the control (Table 5). There was a significant increase (p<0.05) in the mean testes’ luminal diameters of rats in MLT alone and the co-treated groups as compared to the control (Table 5). There was a significant decrease (p<0.05) in the epithelial length of the epididymis of rats treated with DDVP at 24h and 45 days post-treatment compared with the control.

**Table 5:** Testes luminal diameter of rats in treatment groups.

TESTES LUMINAL DIAMETER (µm)	CONTROL	MLT	DDVP	DDVP+MLT
24h post-treatment	244.3±42.53a	252.6±61.05^a^	234.2±39.45b	274.7±46.14c
14 days post-treatment	268.4±62.67^a^	217.2±77.85^a^	210.1±55.88^b^	229.7±53.61^a^
45 days post-treatment	220.5±39.97^a^	231.6±62.94^a^	185±23.72^b^	228.9±33.20^a^

Values are reported as mean ± SD. ^abc^ Means in the same row with different superscript are significant from control (p<0.05).

However, concurrent treatment of DDVP+MLT significantly increased the mean epididymal epithelial length at 24h and 45 days post-treatment (Table 6).

**Table 6:** Epithelial length of the epididymis of rats in treatment groups.

EPITHELIAL LENGTH OF EPIDIDYMIS (µm)	CONTROL	MLT	DDVP	DDVP+MLT
24h post-treatment	21.5±2.028a	18.7±5.764^a^	15.99±3.743b	20.11±4.070^a^
14 days post-treatment	14.59±2.475^a^	17.02±2.129^a^	17.8±3.425^b^	16.6±1.805^a^
45 days post-treatment	15.48±2.601^a^	16.18±3.501^a^	11.74±4.673^b^	11.77±1.332c

Values are reported as mean ± SD. ^abc^ Means in the same row with different superscript are significant from control (p<0.05).

Treatment with MLT caused a significant increase (p<0.05) in the mean epididymal luminal diameter of rats across 24h, 14- and 45-day post-treatment when compared to the control, respectively. There were no significant changes (p<0.05) in the mean epididymal luminal diameter of rats treated with DDVP compared to the control rats except for the significant increase at 45 days post-treatment (Table 7).

**Table 7:** Epididymal luminal diameter of rats in treatment groups.

EPIDIDYMAL LUMINAL DIAMETER (µm)	CONTROL	MLT	DDVP	DDVP+MLT
24h post-treatment	113.1±34.64a	155.1±63.71b	101.5±33.39^a^	122.8±56.56^a^
14 days post-treatment	79.37±23.41^a^	152±62.64^b^	116.2±60.47^a^	199.5±78.36^c^
45 days post-treatment	49.35±9.341^a^	116.2±34.88^b^	104.2±30.63c	116.4±51.71d

Values are reported as mean ± SD. ^abcd^ Means in the same row with different superscript are significant from control (p<0.05).

**Figure 1:**
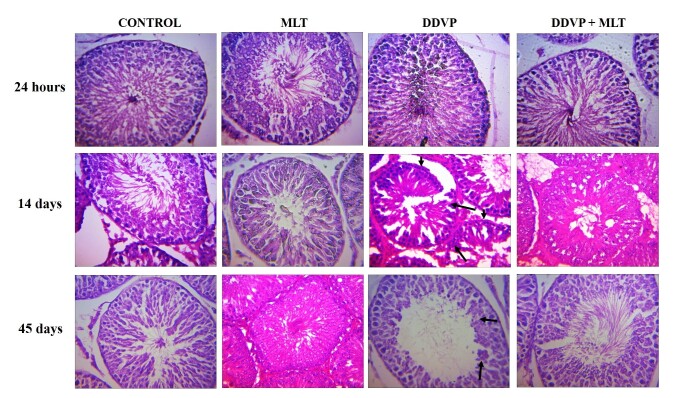
Dichlorvos induced testicular lesions in rats.

**Figure 2:**
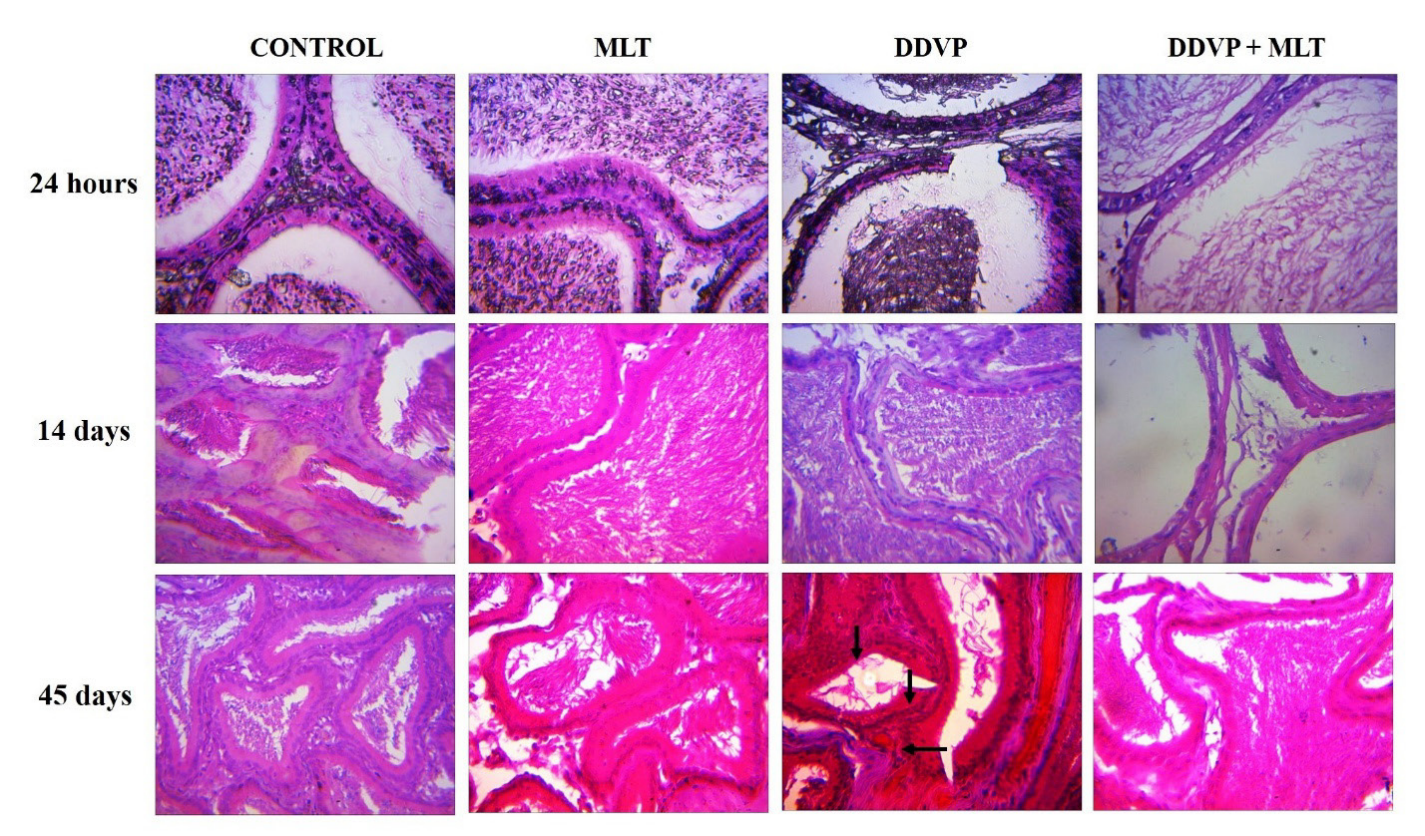
Dichlorvos causes histological lesions in the epididymis of rats after treatment.

Figures 1-2 showed the testicular and epididymal histology of treated rats. There was no lesion observed in the seminiferous tubules of rats’ testes of all the groups in the 24h period of treatments). However, DDVP caused atrophy of the seminiferous tubules and spermatogenic arrest after 14- and 45-day treatments (Figure 1). In addition, all the groups showed normal epithelial tubules and interstitials following 24h and 14 days of treatments. However, DDVP caused atrophy of the epithelial tubules and accentuated interstitial (arrow) after 45-day treatments (Figure 2).

All the groups showed normal seminiferous tubules with normal germinal epithelia and interstitial following 24h treatment. However, DDVP caused atrophy of the seminiferous tubules and spermatogenic arrest (arrow) after 14- and 45-day treatments. A: Control (corn oil), B: MLT-treated group, C: DDVP-treated group; D: DDVP + MLT. Mag. x400 (H&E).

All the groups showed normal epithelial tubules and interstitial following 24h and 14 days treatments. However, DDVP caused atrophy of the epithelial tubules and accentuated interstitial (arrow) after 45 days treatments. A: Control (corn oil), B: MLT-treated group, C: DDVP-treated group; D: DDVP + MLT. Mag. x400 (H&E).

## Discussion

The toxicity of pesticides, such as dichlorvos, has been reported in various body organs, including the male reproductive organs. They distort the lipoidal matrix in cells, generate reactive oxygen species, and promote oxidative stress that interferes with the normal functions of the reproductive system (Joshi et al., 2003; Uzunhisarcikli et al., 2007; Park et al., 2024; Saka et al., 2024; Zhang et al., 2025). However, melatonin functions in defence of environmental stresses via free radical scavenging (Tan et al., 2003; Chitimus et al., 2020) and works to augment the efficiency of other antioxidants (Reiter et al., 2003, Reiter et al., 2016) including the prevention of tissue injury (Jiki et al., 2018; Hu et al., 2023). Therefore, the present study was designed to investigate the protective effects of melatonin on dichlorvos-induced toxicity in testes and epididymis of male Wistar rats.

The observed non-significant changes in the gonadosomatic index of the testes and epididymis across the treatment groups (Tables 1 and 2) support the report by Adamkovicova et al. (2014) and Okamura et al. (2005) in which exposure of rats to dichlorvos did not produce changes in the testes and epididymal weights. On the contrary, dichlorvos caused histopathological changes in the testes and epididymis that have been attributed to androgen deprivation, as seen with other similar toxicants (Dirican and Kalender, 2012; DeGrava and Klinefelter, 2014; Anderson et al., 2020; Akande et al., 2024). This androgen deprivation effect of DDVP resulted in a decrease in the secretory and synthetic functions of the testes and its associated indices, including distorted sperm cells (Anderson et al., 2020). Also, significant androgen deprivation could affect the structure of the epididymis through epithelial apoptosis and reduction in epididymal tubule diameters in all segments of the epididymis (DeGrava and Klinefelter, 2014). We reported in this study the histological changes induced by dichlorvos in both the testes and epididymis (Figures 1-2). Together with the reversal of this effect by melatonin in the co-exposed group, there was also no alteration in the length of the epithelial cells of the epididymis in groups exposed to melatonin (Table 6). This could be linked to the presence of melatonin receptors in the epididymis and testes, which play a significant role in the regulation of epididymal and testicular development, as reported by Duan et al. (2023). Similarly, Deng et al. (2018) reported that melatonin could promote androgen availability in the epididymis and testes since Leydig cells androgen synthesis can be dramatically enhanced by Sertoli cells in the presence of melatonin (Shou-Long et al., 2018).

In addition, MLT alone and its concurrent treatment with DDVP produced a significant increase in the epididymal epithelial length, which may suggest that MLT had no injurious effect on the testes and epididymis of male Wistar rats. This finding and the inability of MLT to induce histological lesions lend support to the documented abilities of MLT to mitigate oxidative stress in tissues (El-Missiry et al., 2007; Othman et al.,2008), with negligible toxicity even in very high doses (El-Sokkary et al., 2005; Reiter et al., 2013). Induction of oxidative stress has been implicated as one of the pathways of the DDVP mechanism of action in experimental animals (Sharma and Singh, 2012). Melatonin in contrast has been reported to scavenge free radicals and prevent the formation of hydrogen peroxide, which is one of the most damaging oxidants, thereby acting as a Type I and Type II antioxidant. (Galano et al., 2018).

The histomorphometric assessment of the testes showed that MLT caused a significant increase in the testes and epididymal luminal diameter when used alone and in the co-exposed group. This implies that melatonin serves as a therapeutic agent that can improve spermatogenesis since increased tested and epididymal luminal diameters have been reported to be associated with enhanced sperm production and fertility (Parlaktas et al., 2014). In addition, the study conducted by Yurtcu et al. (2009) showed the effect of melatonin dosages on the testicular architecture. The authors reported that a single dose of MLT was unable to alter the structure of the testes and Johnsen’s score; whereas 7 doses improved the testis's structure. In contrast, the report of Mirhoseini et al. (2017) claimed that the administration of MLT at 25 µg/kg did not significantly increase testes diameter and therefore, the increase is negligible. However, this might be a result of the testicular damage induced by acute unilateral spermatic cord torsion and detorsion in their experimental rats. Therefore, it can be suggested that testicular torsion can negatively affect the therapeutic role of melatonin in increasing testicular and epididymal luminal diameters. However, there were no significant differences in the epididymal luminal diameter in the DDVP-treated group, except for the significant spike in the chronic stage. This finding is consistent with the report of Faris (2018) who reported that the administration of 0.1 mg/kg and 0.05 mg/kg of dichlorvos for 15 days does not show significant differences in the luminal diameter of testes rats. We hypothesised that this could be a result of exposing the experimental rats to low doses of dichlorvos.

## Conclusion

We have shown in this study that melatonin and dichlorvos did not alter the gonadosomatic index, epididymal length, and diameter of the testes and epididymis. However, dichlorvos alters the architecture of the epididymis, which was restored by melatonin in the co-treated group. In addition, we have suggested that melatonin would be effective in enhancing spermatogenesis and sperm storage and maturation due to the increased testicular and epididymal luminal diameters.
